# Medical Students as Nurses—I

**Published:** 1907-11-16

**Authors:** 


### MEDICAL STUDENTS AS NURSES.—I.

We have often noticed that some confusion of thought exists in regard to the knowledge necessary for a doctor and the knowledge necessary for a nurse. It is usually assumed that doctors know all about nursing, although a very little reflection will make it evident that this is not the case. The art of nursing has reached its present stage chiefly by the aid of intelligent women, who, for the last two or three generations, have devoted their energies, their abilities and their attention to rendering skilled services to the sick. They have studied the best means of relieving suffering by ascertaining the most intelligent way in which the treatment ordered by the doctor can be administered, and they have handed on to others the methods which they have found the most satisfactory in their own experience. Thus a "trained" way of carrying out treatment ordered by the doctor has become generally recognised, and a skilled manner of performing minor attentions required by the sick is now universally associated with the idea of trained nursing. In earlier days especially, nurses have been helped to a better understanding of their duties by lectures from doctors and this is still the case, but such lectures can only constitute a small part of their actual training.

The knowledge of a medical man not only covers wider ground, but is different in kind and in degree to the knowledge necessary for a nurse. Both are concerned with the care of sick people, but while it is the doctor's business to make the correct diagnosis and to order the treatment for the case, the carrying out of that treatment chiefly depends upon the nurse. Her practical experience in administering the treatment ordered very largely exceeds that of the doctor, and experienced medical men are not only ready to concede that this is the case, but are often wisely desirous of pointing out to their juniors the wisdom of seeking definite information in this connection from sisters and nurses, knowing that they will find it invaluable in private practice later on. The need for a doctor to familiarise himself with the best methods of carrying out nursing details is often not apparent until some unpleasant experience in private practice makes him realise that the fact of always having had his orders carried out efficiently under skilled supervision in the wards of the hospital has induced the belief that the treatment he prescribed for his patient would be carried out automatically with ease and accuracy. On many occasions a general practitioner's desire to have some important remedy applied will find him without any one at hand capable of doing what he has hitherto regarded as quite a simple matter, or else he will find willing though totally inexperienced helpers ready to do all in their power, but waiting to receive minute information from him as to how the simplest remedies are to be prepared and applied. The range of study which leads a doctor to decide that a certain treatment is necessary, and to hope that certain results will ensue from the efficient application of that treatment, does not necessarily include the best methods of applying it, though it is too readily assumed that this is the case. This carrying out of treatment is essentially the nurse's province, and this is where the value of her skilled assistance comes in.

Accurate elementary knowledge on various purely medical subjects is a distinct help towards intelligent nursing. Careful grounding in the earlier days of a nurse's training, in the reasons why certain remedies are applied, is essential if the treatment is to be carried out with skill and intelligence. This is the knowledge which doctors can impart to nurses, by lectures and demonstrations. But it is the exceptional doctor only who is able to keep the nurse's point of view before him, and to be guided by it in his selection of the knowledge he imparts, so that her nursing is rendered of greater assistance to himself, and therefore of greater value to the patient.

But then we come to the other side of the question. Trained nurses spend their days and nights not only in watching over patients, but in constantly doing something for them. "Give this remedy" and "apply that treatment" are the doctor's orders to the nurse, and she, having been taught herself in the probationer days the most approved methods of carrying out such instructions, has by long and repeated practice gained facility in executing these orders, and in tending on sick people in skilled ways. She can ensure that the doctor's orders are efficiently carried out, so that his intention may be fulfilled, and she can also add to the comfort and tend to promote the relief of the patient by every means that knowledge, skill and practical experience can devise. Our leading physicians and surgeons never hesitate to impress upon their students how much they themselves owe to the sisters or nurses with whom they were associated in their own student days. But too often it has to be admitted that the doctor's need for this nursing knowledge—*i.e.*, of how best to carry out with practical efficiency the treatment he wishes applied—has been discovered by some experience when his own ignorance proved very inconvenient for the time being. The senior physician or surgeon, going the round of his ward, has come to realise from his own experience outside the hospital how much patients suffer without trained nursing, and how sadly the skill he is ready to exercise on their behalf can be frustrated by ignorant or inefficient nursing. Experience has taught him the value of skilled nursing, both by the gratifying results of it which he has seen in and outside the hospital, and by the misery and suffering arising from the lack of it on other occasions. It is this experience which enables him to estimate the care and attention which is connected with the absence of bedsores in cases where it is almost a miracle that they do not exist, and the gracious words of praise to this effect, so often given to the sister (rather to the surprise of the students who, so far, have had no occasion to concern themselves with such matters), are regarded by sister and nurse as the greatest encouragement, and an ample reward for all the care and trouble bestowed for many weeks to maintain a good result.

*(To be continued.)*

